# Supplementary figures and images for: Neutrophil extracellular traps in the animal model of adenine-induced chronic kidney disease
Source: PLoS One. 2026 Jun 5;21(6):e0350004. doi: 10.1371/journal.pone.0350004 (PMC13240914; doi:10.1371/journal.pone.0350004)

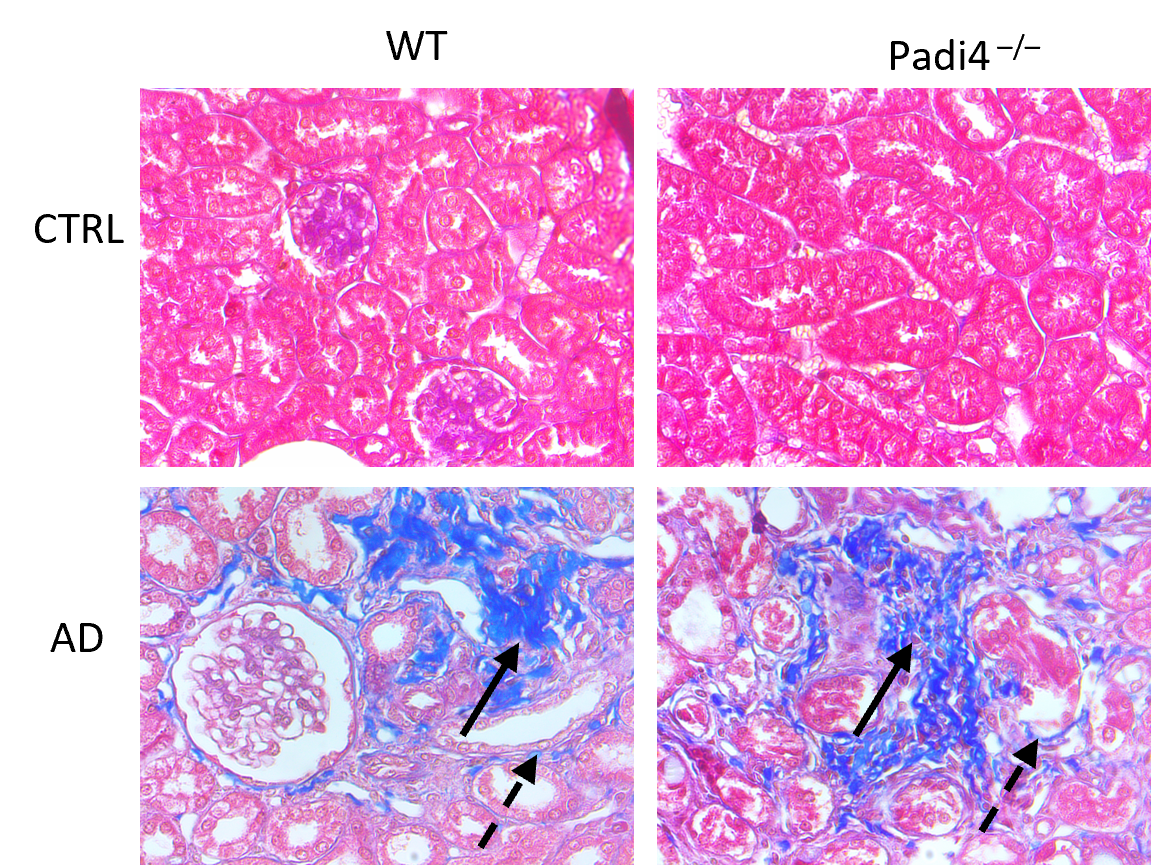

Supplement: S1 Fig — Picro Mallory Trichrome staining was used to assess renal fibrosis visualized by blue colour as well as tubular dilatation. An abundant interstitial fibrosis and tubular dilatation was found in both adenine-treated groups (WT and Padi4−/−; black arrow and dashed arrows, respectively), whereas no fibrosis and no tubular dilatation was found in both control saline-treated groups (WT and Padi4−/−). Images were cached at a magnification of 400 x. (TIF) [file pone.0350004.s001.tif]

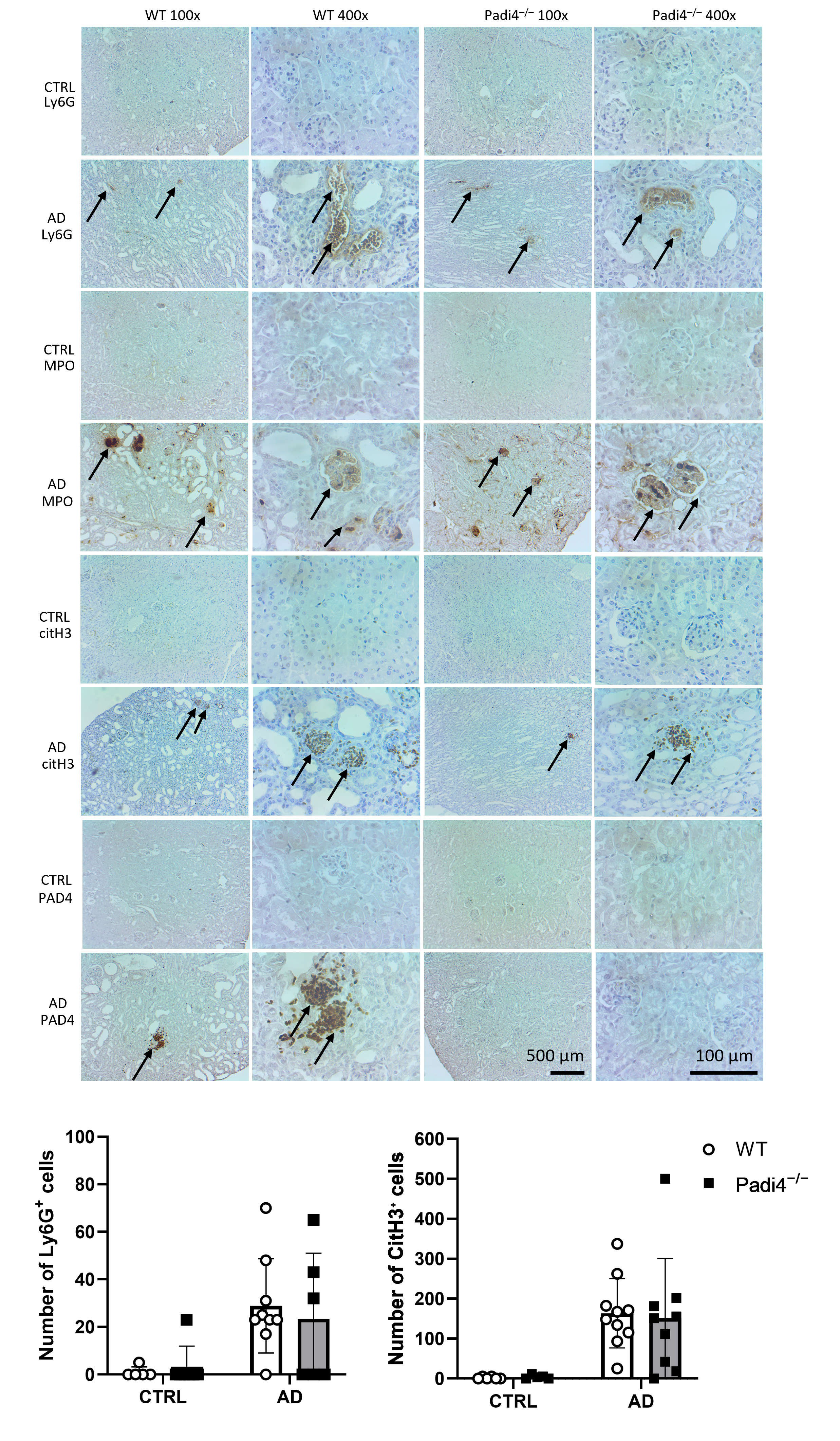

Supplement: S2 Fig — Ly6G, MPO, citH3, and PAD4 immunostaining were performed on sections from all four groups of mice. Ly6G expression was found in both genotypes treated with adenine, whereas no expression was found in both control groups. Moreover, abundant MPO expression was found in both, interstitium and glomeruli in adenine-treated groups of both genotypes, whereas no expression was found in both control groups. Additionally, citH3 expression was found in tubuli of both genotypes treated with adenine, whereas no or little expression was found in both control groups. PAD4 staining was found in adenine-treated WT, whereas no positive staining was found in Padi4 −/− mice with and without adenine treatment, as well as in WT mice without adenine treatment. Images were cached at a magnification of × 100 and × 400. Dark brown spots revealed positive staining. AD – adenine-treated group, CTRL – control group receiving saline, citH3 – citrullinated histone H3, Ly6G – lymphocyte antigen 6 complex locus G, MPO – myeloperoxidase, Padi4−/−– protein-arginine deiminase type 4 knockout mice, WT – wild-type mice. (TIF) [file pone.0350004.s002.tif]
