# Supplementary material for: Neutrophil extracellular traps in the animal model of adenine-induced chronic kidney disease
Source: PLoS One. 2026 Jun 5;21(6):e0350004. doi: 10.1371/journal.pone.0350004 (PMC13240914; doi:10.1371/journal.pone.0350004)
Supplement: S1 Table — Values p ≤ 0.05 are considered statistically significant. (DOCX) [file pone.0350004.s003.docx]

**S1 Table**. **Relation between NET-associated markers in plasma and in urine of all animals, regardless of genotype or treatment.**

|  | **Plasma ecDNA** | **Plasma MPO** | **Plasma NE** | **Plasma NGAL** | **Urine ecDNA** | **Urine MPO** | **Urine NE** | **Urine NGAL** |
| --- | --- | --- | --- | --- | --- | --- | --- | --- |
| **Plasma ecDNA** |  | r=0.21  p<0.05 | r=0.28  p<0.01 | r=0.27  p<0.05 | r=0.27  p<0.05 | r= -0.06  p>0.05 | r= -0.04  p>0.05 | r=0.27  p<0.05 |
| **Plasma MPO** | r=0.21  p<0.05 |  | r=0.80  p<0.001 | r=0.75  p<0.001 | r=0.30  p<0.05 | r=0.06  p>0.05 | r=0.03  p>0.05 | r=0.55  p<0.001 |
| **Plasma NE** | r=0.28  p<0.01 | r=0.80  p<0.001 |  | r=0.85  p<0.001 | r=0.47  p<0.001 | r=0.08  p>0.05 | r=0.12  p>0.05 | r=0.69  p<0.001 |
| **Plasma NGAL** | r=0.27  p<0.05 | r=0.75  p<0.001 | r=0.85  p<0.001 |  | r=0.54  p<0.001 | r=0.07  p>0.05 | r=0.18  p>0.05 | r=0.79  p<0.001 |
| **Urine ecDNA** | r=0.27  p<0.05 | r=0.30  p<0.05 | r=0.47  p<0.001 | r=0.54  p<0.001 |  | r=0.58  p<0.001 | r=0.24  p=0.10 | r=0.27  p<0.05 |
| **Urine MPO** | r= -0.06  p>0.05 | r=0.06  p>0.05 | r=0.08  p>0.05 | r=0.07  p>0.05 | r=0.58  p<0.001 |  | r=0.24  p<0.05 | r= -0.13  p>0.05 |
| **Urine NE** | r= -0.04  p>0.05 | r=0.03  p>0.05 | r=0.12  p>0.05 | r=0.18  p>0.05 | r=0.24  p=0.10 | r=0.24  p<0.05 |  | r=0.08  p>0.05 |
| **Urine NGAL** | r=0.27  p<0.05 | r=0.55  p<0.001 | r=0.69  p<0.001 | r=0.79  p<0.001 | r=0.27  p<0.05 | r= -0.13  p>0.05 | r=0.08  p>0.05 |  |

ecDNA – extracellular DNA, MPO – myeloperoxidase, NE – neutrophil elastase, NGAL – neutrophil gelatinase-associated lipocalin, r – Spearman´s rank-order correlation coefficient. Values p ≤ 0.05 are considered statistically significant.
