# Supplementary material for: Neutrophil extracellular traps in the animal model of adenine-induced chronic kidney disease
Source: PLoS One. 2026 Jun 5;21(6):e0350004. doi: 10.1371/journal.pone.0350004 (PMC13240914; doi:10.1371/journal.pone.0350004)
Supplement: S1 Methods — (DOCX) [file pone.0350004.s004.docx]

*Histology*

Picro Mallory Trichrome staining was performed to visualize fibrosis, with a blue stain of collagen. Kidneys were cut into 4 μm paraffin sections, dewaxed and processed according to the manufacturer’s protocol. The representative images were cached at a magnification of × 400.

*Immunohistochemistry*

Immunohistochemistry for Ly-6G (Cell Signaling, 87048), MPO (Abcam, ab208670), citH3 (Abcam, ab52946), and PAD4 (Abcam, ab214810) was performed on kidneys taken at the end of the experiment. Kidneys were cut into 3 μm paraffin sections, dewaxed and subjected to antigen retrieval by 15 minutes incubation in 10 mM citrate buffer, pH 6.0, at 80 °C (Ly6G and citH3) or 10 mM TRIS/EDTA pH 9 (MPO and PAD4). The concentrations of antibodies were 1:100, 1:100, 1:200, and 1:200 in 1%BSA/PBS, respectively. For all proteins, a 2-step immunoperoxidase technique was used according to standard techniques. Peroxidase activity was developed using diaminobenzidine and H_2_O_2_. The representative images were cached at a magnification of × 100 and × 400. Ten fields per one kidney slice were evaluated by an independent scientist (cell count for Ly6G and citH3, positive areas for MPO, PAD4).
